# Predictors of early remission of proteinuria in adult patients with minimal change disease: a retrospective cohort study

**DOI:** 10.1038/s41598-022-13067-7

**Published:** 2022-06-13

**Authors:** Ryohei Yamamoto, Enyu Imai, Shoichi Maruyama, Hitoshi Yokoyama, Hitoshi Sugiyama, Asami Takeda, Shunya Uchida, Tatsuo Tsukamoto, Kazuhiko Tsuruya, Yasuhiro Akai, Kosaku Nitta, Megumu Fukunaga, Hiroki Hayashi, Kosuke Masutani, Takashi Wada, Tsuneo Konta, Ritsuko Katafuchi, Saori Nishio, Shunsuke Goto, Hirofumi Tamai, Arimasa Shirasaki, Tatsuya Shoji, Kojiro Nagai, Tomoya Nishino, Kunihiro Yamagata, Junichiro J. Kazama, Keiju Hiromura, Hideo Yasuda, Makoto Mizutani, Tomohiko Naruse, Takeyuki Hiramatsu, Kunio Morozumi, Hiroshi Sobajima, Yosuke Saka, Eiji Ishimura, Daisuke Ichikawa, Takashi Shigematsu, Tadashi Sofue, Shouichi Fujimoto, Takafumi Ito, Hiroshi Sato, Ichiei Narita, Yoshitaka Isaka, JNSCS Investigators, Saori Nishio, Yasunobu Ishikawa, Daigo Nakazawa, Tasuku Nakagaki, Toshinobu Sato, Mitsuhiro Sato, Satoru Sanada, Hiroshi Sato, Mariko Miyazaki, Takashi Nakamichi, Tae Yamamoto, Kaori Narumi, Gen Yamada, Tsuneo Konta, Kazunobu Ichikawa, Junichiro James Kazama, Tsuyoshi Watanabe, Koichi Asahi, Yuki Kusano, Kimio Watanabe, Kunihiro Yamagata, Joichi Usui, Shuzo Kaneko, Tetsuya Kawamura, Keiju Hiromura, Akito Maeshima, Yoriaki Kaneko, Hidekazu Ikeuchi, Toru Sakairi, Masao Nakasatomi, Hajime Hasegawa, Takatsugu Iwashita, Taisuke Shimizu, Koichi Kanozawa, Tomonari Ogawa, Kaori Takayanagi, Tetsuya Mitarai, Hirokazu Okada, Tsutomu Inoue, Hiromichi Suzuki, Kouji Tomori, Kosaku Nitta, Takahito Moriyama, Akemi Ino, Masayo Sato, Shunya Uchida, Hideaki Nakajima, Hitoshi Homma, Nichito Nagura, Yoshifuru Tamura, Shigeru Shibata, Yoshihide Fujigaki, Yusuke Suzuki, Yukihiko Takeda, Isao Osawa, Teruo Hidaka, Daisuke Ichikawa, Yugo Shibagaki, Sayuri Shirai, Tsutomu Sakurada, Tomo Suzuki, Mikako Hisamichi, Ichiei Narita, Naohumi Imai, Yumi Ito, Shin Goto, Yoshikatsu Kaneko, Rhohei Kaseda, Hitoshi Yokoyama, Keiji Fujimoto, Norifumi Hayashi, Takashi Wada, Miho Shimizu, Kengo Furuichi, Norihiko Sakai, Yasunori Iwata, Tadashi Toyama, Shinji Kitajima, Kiyoki Kitagawa, Hiroshi Sobajima, Norimi Ohashi, So Oshitani, Kiyohito Kawashima, Tetsushi Mimura, Hideo Yasuda, Akira Hishida, Yoshihide Fujigaki, Satoshi Tanaka, Noriko Mori, Toshiyuki Akahori, Yutaka Fujita, Shoichi Maruyama, Naotake Tsuboi, Tomoki Kosugi, Takuji Ishimoto, Takayuki Katsuno, Noritoshi Kato, Waichi Sato, Asami Takeda, Kunio Morozumi, Yasuhiro Ohtsuka, Hibiki Shinjo, Akihito Tanaka, Hiroki Hayashi, Yukio Yuzawa, Midori Hasegawa, Daijo Inaguma, Shigehisa Koide, Kazuo Takahashi, Takeyuki Hiramatsu, Shinji Furuta, Hideaki Ishikawa, Hirofumi Tamai, Takatoshi Morinaga, Arimasa Shirasaki, Toshiki Kimura, Mina Kato, Shizunori Ichida, Nobuhide Endo, Tomohiko Naruse, Yuzo Watanabe, Yosuke Saka, Satashi Suzuki, Michiko Yamazaki, Rieko Morita, Kunio Morozumi, Kunio Morozumi, Kaoru Yasuda, Chika Kondo, Takahiro Morohiro, Rho Sato, Yuichi Shirasawa, Yoshiro Fujita, Hideaki Shimizu, Tatsuhito Tomino, Makoto Mizutani, Yosuke Saka, Hiroshi Nagaya, Makoto Yamaguchi, Tatsuo Tsukamoto, Eri Muso, Hiroyuki Suzuki, Tomomi Endo, Hiroko Kakita, Megumu Fukunaga, Tatsuya Shoji, Terumasa Hayashi, Eiji Ishimura, Akihiro Tsuda, Shinya Nakatani, Ikue Kobayashi, Mitsuru Ichii, Akinobu Ochi, Yoshiteru Ohno, Yoshitaka Isaka, Enyu Imai, Yasuyuki Nagasawa, Hirotsugu Iwatani, Ryohei Yamamoto, Tomoko Namba, Shunsuke Goto, Shinichi Nishi, Yasuhiro Akai, Ken-ichi Samejima, Masaru Matsui, Miho Tagawa, Kaori Tanabe, Hideo Tsushima, Takashi Shigematsu, Masaki Ohya, Shigeo Negi, Toru Mima, Takafumi Ito, Hitoshi Sugiyama, Keiko Tanaka, Toshio Yamanari, Masashi Kitagawa, Akifumi Onishi, Koki Mise, Naoki Kashihara, Tamaki Sasaki, Sohachi Fujimoto, Hajime Nagasu, Kojiro Nagai, Toshio Doi, Tadashi Sofue, Hideyasu Kiyomoto, Kumiko Moriwaki, Taiga Hara, Yoko Nishijima, Yoshio Kushida, Tetsuo Minamino, Yoshio Terada, Taro Horino, Yoshinori Taniguchi, Kosuke Inoue, Yoshiko Shimamura, Tatsuki Matsumoto, Kazuhiko Tsuruya, Hisako Yoshida, Naoki Haruyama, Shunsuke Yamada, Akihiro Tsuchimoto, Yuta Matsukuma, Kosuke Masutani, Yasuhiro Abe, Aki Hamauchi, Tetsuhiko Yasuno, Kenji Ito, Kei Fukami, Junko Yano, Chika Yoshida, Yuka Kurokawa, Nao Nakamura, Ritsuko Katafuchi, Hiroshi Nagae, Shumei Matsueda, Kazuto Abe, Tomoya Nishino, Tadashi Uramatsu, Yoko Obata, Shouichi Fujimoto, Yuji Sato, Masao Kikuchi, Ryuzo Nishizono, Takashi Iwakiri, Hiroyuki Komatsu

**Affiliations:** 1 Health and Counseling Center, Osaka University, 1-17 Machikaneyama-cho, Toyonaka, Osaka 560-0043 Japan; 2 Department of Nephrology, Osaka University Graduate School of Medicine, 2-2-D11 Yamadaoka, Suita, Osaka 565-0871 Japan; 3 Nakayamadera Imai Clinic, 2-8-18 Nakayamadera, Takarazuka, Hyogo 665-0861 Japan; 4 Department of Nephrology, Nagoya University Graduate School of Medicine, 65 Tsurumai-cho, Showa-ku, Nagoya, Aichi 466-8550 Japan; 5 Department of Nephrology, Kanazawa Medical University School of Medicine, 1-1 Daigaku, Uchinada, Kahoku, Ishikawa 920-0293 Japan; 6 Department of Nephrology, Rheumatology, Endocrinology and Metabolism, Okayama University Graduate School of Medicine, Dentistry and Pharmaceutical Sciences, 2-5-1 Shikatacho, Kita-ku, Okayama, Okayama 700-8558 Japan; 7 Kidney Disease Center, Japanese Red Cross Nagoya Daini Hospital, 2-9 Myokencho, Showa-ku, Nagoya, Aichi 466-8650 Japan; 8 Department of Internal Medicine, Teikyo University School of Medicine, 2-11-1 Kaga, Itabashi-ku, Tokyo, 173-8606 Japan; 9 Department of Nephrology and Dialysis, Kitano Hospital, Tazuke Kofukai Medical Research Institute, 2-4-20 Ogimachi, Kita-ku, Osaka, Osaka 530-8480 Japan; 10 Department of Nephrology, Nara Medical University, 840 Shijo-cho, Kashihara, Nara 634-8521 Japan; 11 First Department of Internal Medicine, Nara Medical University, 840 Shijocho, Kashihara, Nara 634-8522 Japan; 12 Department of Nephrology, Tokyo Women’s Medical University, 8-1 Kawada-cho, Shinjuku-ku, Tokyo, 162-8666 Japan; 13 Division of Nephrology, Department of Internal Medicine, Toyonaka Municipal Hospital, 4-14-1 Shibaharacho, Toyonaka, Osaka 560-8565 Japan; 14 Department of Nephrology, Fujita Health University School of Medicine, 1-98 Dengakugakubo, Kutsukakecho, Toyoake, Aichi 470-1192 Japan; 15 Division of Nephrology and Rheumatology, Department of Internal Medicine, Faculty of Medicine, Fukuoka University, 7-45-1 Nanakuma, Jonan-ku, Fukuoka, Fukuoka 814-0180 Japan; 16 Department of Nephrology and Laboratory Medicine, Kanazawa University, 13-1 Takara-machi, Kanazawa, Ishikawa 920-8641 Japan; 17 Department of Cardiology, Pulmonology, and Nephrology, Yamagata University School of Medicine, 2-2 Iida-Nishi, Yamagata-shi, Yamagata, Yamagata 990-9585 Japan; 18 Kidney Unit, National Hospital Organization Fukuokahigashi Medical Center, 1-1-1 Chidori, Koga, Fukuoka 811-3195 Japan; 19 Division of Rheumatology, Endocrinology and Nephrology, Hokkaido University Graduate School of Medicine, Kita 15, Nishi 7, Kita-ku, Sapporo, Hokkaido 060-8638 Japan; 20 Division of Nephrology and Kidney Center, Kobe University Graduate School of Medicine, 7-5-1 Kusunokicho, Chuo-ku, Kobe, Hyogo 650-0017 Japan; 21 Department of Nephrology, Anjo Kosei Hospital, 28 Higashihirokute, Anjocho, Anjo, Aichi 446-8602 Japan; 22 Department of Nephrology, Ichinomiya Municipal Hospital, 2-2-22 Bunkyo, Ichinomiya, Aichi 491-8558 Japan; 23 Department of Kidney Disease and Hypertension, Osaka General Medical Center, 3-1-56 Bandaihigashi, Sumiyoshi-ku, Osaka, Osaka 558-8558 Japan; 24 Department of Nephrology, Institute of Biomedical Sciences, Tokushima University Graduate School, 3-18-15, Kuramoto-cho, Tokushima, 770-8503 Japan; 25 Department of Nephrology, Nagasaki University Hospital, 1-7-1 Sakamoto, Nagasaki, Nagasaki 852-8501 Japan; 26 Department of Nephrology, Faculty of Medicine, University of Tsukuba, 1-1-1 Tennodai, Tsukuba, Ibaraki 305-8575 Japan; 27 Department of Nephrology and Hypertension, Fukushima Medical University School of Medicine, 1 Hikariga-oka, Fukushima-City, Fukushima 960-1295 Japan; 28 Department of Nephrology and Rheumatology, Gunma University Graduate School of Medicine, 3-39-22 Showa, Maebashi, Gunma 371-8511 Japan; 29 Internal Medicine 1, Hamamatsu University School of Medicine, 1-20-1 Handayama, Higashi-ku, Hamamatsu, Shizuoka 431-3192 Japan; 30 Department of Nephrology, Handa City Hospital, 2-29 Toyocho, Handa, Aichi 475-8599 Japan; 31 Department of Nephrology, Kasugai Municipal Hospital, 1-1-1 Takakicho, Kasugai, Aichi 486-8510 Japan; 32 Department of Nephrology, Konan Kosei Hospital, 137 Omatsubara, Takayacho, Konan, Aichi 483-8704 Japan; 33 Department of Nephrology, Masuko Memorial Hospital, 35-28 Takebashicho, Nakamura-ku, Nagoya, Aichi 453-8566 Japan; 34 Department of Diabetology and Nephrology, Ogaki Municipal Hospital, 4-86 Minaminokawacho, Ogaki, Gifu 503-8502 Japan; 35 Department of Nephrology, Yokkaichi Municipal Hospital, 2-2-37 Shibata, Yokkaichi, Mie 510-8567 Japan; 36 Department of Nephrology, Osaka City University Graduate School of Medicine, 1-4-3 Asahimachi, Abeno-ku, Osaka, 545-8585 Japan; 37 Division of Nephrology and Hypertension, Department of Internal Medicine, St. Marianna University School of Medicine, 2-16-1 Sugao, Miyamae-ku, Kawasaki, Kanagawa 216-000 Japan; 38 Department of Nephrology, Wakayama Medical University, 811-1 Kimiidera, Wakayama-City, Wakayama 641-8509 Japan; 39 Department of Cardiorenal and Cerebrovascular Medicine, Kagawa University, 1750-1 Ikenobe, Miki-cho, Kita-gun, Kagawa 761-0793 Japan; 40 Department of Hemovascular Medicine and Artificial Organs, Faculty of Medicine, University of Miyazaki, 5200 Kihara, Kiyotakecho, Miyazaki, Miyazaki 889-1692 Japan; 41 Division of Nephrology, Shimane University Hospital, 89-1 Enyacho, Izumo, Shimane 693-8501 Japan; 42 Department of Nephrology, Endocrinology, and Vascular Medicine, Tohoku University Graduate School of Medicine, 1-1 Seiryo-cho, Aoba-ku, Sendai, Miyagi 980-8574 Japan; 43 Division of Clinical Nephrology and Rheumatology, Kidney Research Center, Niigata University Graduate School of Medical and Dental Sciences, 757 Ichibancho, Asahimachi-dori, Chuo Ward, Niigata, Niigata 951-8510 Japan; 44 Hokkaido University Hospital, Sapporo, Hokkaido Japan; 45 JCHO Sendai Hospital, Sendai, Miyagi Japan; 46 Tohoku University Hospital, Sendai, Miyagi Japan; 47 Yamagata University Hospital, Yamagata, Yamagata Japan; 48 Fukushima Medical University Hospital, Fukushima, Fukushima Japan; 49 University of Tsukuba Hospital, Tsukuba, Ibaraki Japan; 50 Gunma University Hospital, Maebashi, Gunma Japan; 51 Saitama Medical Center, Saitama Medical University, Kawagoe, Saitama Japan; 52 Department of Nephrology, Saitama Medical University, Irumagun, Saitama Japan; 53 Tokyo Women’s Medical University, Shinjuku-ku, Tokyo, Japan; 54 Teikyo University School of Medicine, Itabashi-ku, Tokyo, Japan; 55 Juntendo Faculty of Medicine, Bunkyo-ku, Tokyo, Japan; 56 St. Marianna University, Kawasaki, Kanagawa Japan; 57 Niigata University Medical and Dental Hospital, Niigata, Niigata Japan; 58 Kanazawa Medical University, Uchinada, Ishikawa Japan; 59 Kanazawa University Hospital, Kanazawa, Ishikawa Japan; 60 National Hospital Organization Kanazawa Medical Center, Kanazawa, Ishikawa Japan; 61 Ogaki Municipal Hospital, Ogaki, Gifu Japan; 62 Gifu Prefectural Tajimi Hospital, Tajimi, Gifu Japan; 63 Hamamatsu University Hospital, Hamamatsu, Shizuoka Japan; 64 Shizuoka General Hospital, Shizuoka, Shizuoka Japan; 65 Chutoen General Medical Center, Kakegawa, Shizuoka Japan; 66 Nagoya University Graduate School of Medicine, Nagoya, Aichi Japan; 67 Japanese Red Cross Nagoya Daini Hospital, Nagoya, Aichi Japan; 68 Fujita Health University School of Medicine, Toyoake, Aichi Japan; 69 Konan Kosei Hospital, Konan, Aichi Japan; 70 Anjo Kosei Hospital, Anjo, Aichi Japan; 71 Ichinomiya Municipal Hospital, Ichinomiya, Aichi Japan; 72 Japanese Red Cross Nagoya Daiichi Hospital, Nagoya, Aichi Japan; 73 Kasugai Municipal Hospital, Kasugai, Aichi Japan; 74 Kainan Hospital, Yatomi, Aichi Japan; 75 Masuko Memorial Hospital, Nagoya, Aichi Japan; 76 Chubu Rosai Hospital, Nagoya, Aichi Japan; 77 Handa City Hospital, Handa, Aichi Japan; 78 Yokkaichi Municipal Hospital, Yokkaichi, Mie Japan; 79 Kitano Hospital, Osaka, Osaka Japan; 80 Toyonaka Municipal Hospital, Toyonaka, Osaka Japan; 81 Osaka General Medical Center, Osaka, Osaka Japan; 82 Osaka City University Hospital, Osaka, Osaka Japan; 83 Osaka University Hospital, Suita, Osaka Japan; 84 Kobe University Hospital, Kobe, Hyogo Japan; 85 Nara Medical University Hospital, Kashihara, Nara Japan; 86 Wakayama Medical University Hospital, Wakayama, Wakayama Japan; 87 Shimane University Hospital, Izumo, Shimane Japan; 88 Okayama University Hospital, Okayama, Okayama Japan; 89 Kawasaki Medical School, Kurashiki, Okayama Japan; 90 Graduate School of Medicine, The University of Tokushima, Tokushima, Tokushima Japan; 91 Kagawa University, Miki-cho, Takamatsu, Japan; 92 Kochi Medical School, Kochi University, Nankoku, Kochi Japan; 93 Kyushu University Hospital, Fukuoka, Fukuoka Japan; 94 Fukuoka University Hospital, Fukuoka, Fukuoka Japan; 95 Kurume University Hospital, Kurume, Fukuoka Japan; 96 National Hospital Organization Fukuokahigashi Medical Center, Koga, Fukuoka Japan; 97 Nagasaki University Hospital, Nagasaki, Nagasaki Japan; 98 Miyazaki University Hospital, Miyazaki, Miyazaki Japan

**Keywords:** Minimal change disease, Epidemiology

## Abstract

Previous studies reported conflicting results regarding an association between serum albumin concentration and the cumulative incidence of remission of proteinuria in adult patients with minimal change disease (MCD). The present study aimed to clarify the clinical impact of serum albumin concentration and the cumulative incidence of remission and relapse of proteinuria in 108 adult patients with MCD at 40 hospitals in Japan, who were enrolled in a 5-year prospective cohort study of primary nephrotic syndrome, the Japan Nephrotic Syndrome Cohort Study (JNSCS). The association between serum albumin concentration before initiation of immunosuppressive treatment (IST) and the cumulative incidence of remission and relapse were assessed using multivariable-adjusted Cox proportional hazards models. Remission defined as urinary protein < 0.3 g/day (or g/gCr) was observed in 104 (96.3%) patients. Of 97 patients with remission within 6 month of IST, 42 (43.3%) developed relapse defined as ≥ 1.0 g/day (or g/gCr) or dipstick urinary protein of ≥ 2+. Serum albumin concentration was significantly associated with remission (multivariable-adjusted hazard ratio [95% confidence interval] per 1.0 g/dL, 0.57 [0.37, 0.87]), along with eGFR (per 30 mL/min/1.73 m^2^: 1.43 [1.08, 1.90]), whereas they were not associated with relapse. A multivariable-adjusted model showed that patients with high eGFR level (≥ 60 mL/min/1.73 m^2^) and low albumin concentration (≤ 1.5 g/dL) achieved significantly early remission, whereas those with low eGFR (< 60 mL/min/1.73 m^2^) and high albumin concentration (> 1.5 g/dL) showed significantly slow remission. In conclusion, lower serum albumin concentration and higher eGFR were associated with earlier remission in MCD, but not with relapse.

## Introduction

Minimal change disease (MCD) is one of the major primary nephrotic syndromes^1–3^. MCD in adults is highly steroid-sensitive, but steroid resistance is seen in 5–20% of adult patients with MCD^4^. Epidemiological studies have showed that the incidence of end-stage kidney disease (ESKD) is remarkably lower in patients with MCD than in those with MN and FSGS^5,6^, concluding that MCD typically has favorable outcomes. However, compared with the general population, patients with MCD were at significantly higher risk of ESKD and thromboembolism^7^. Because steroid resistance predicts the incidence of ESKD in adult patients with MCD^8^, clinical characteristics associated with steroid sensitivity should be clarified to stratify the patients with MCD into several groups with different levels of steroid sensitivity.

Early small retrospective cohort studies published between the 1980s and the 2000s including ≤ 62 adult patients with MCD, suggested that several clinical factors were associated with steroid sensitivity in adult patients with MCD without controlling for potential clinical confounders, including age^9,10^, serum concentrations of creatinine^11^ and albumin^10^, selectivity index of proteinuria^11^, microscopic hematuria^11^, and acute kidney injury (AKI)^12^. However, their results may be biased without controlling for potential confounding factors. Recent Japanese retrospective cohort studies, including 142^13^ or 125^14^ patients aged ≥ 15 years with MCD, confirmed that young age^13,14^, low serum creatinine concentration^13,14^, and low urinary protein level^14^ independently predicted early remission, even after adjusting for clinically relevant factors. Another Japanese retrospective cohort study identified low serum albumin concentration, not urinary protein level, as a significant predictor of early remission, besides young age and no AKI^15^. The findings of these two studies strongly suggest that high glomerular filtration rate (GFR) level is a predictor of early remission. In contrast, the impacts of urinary protein level and serum albumin concentration on remission were conflicting, which should be examined in a multicenter cohort study with external validity.

The aim of the present cohort study was to identify the clinical predictors of remission and relapse of proteinuria in adult patients with MCD, with great interest in serum albumin concentration, urinary protein level, and GFR. We used the clinical data collected prospectively in 108 adult patients with MCD in 40 hospitals, who were enrolled in a 5-year prospective cohort study, the Japan Nephrotic Syndrome Cohort Study (JNSCS)^16–20^. The results of the present study provide useful clinical information to identify patients at a high risk of steroid resistance, who might need intensive immunosuppressive therapy (IST).

## Results

Clinical characteristics of 108 adult patients with MCD included in the present study were listed in Table 1. Medina age was 43 years (interquartile range 30, 64) and 61.1% were male patients. Numbers (proportions) of the patients with eGFR of < 30, 30–59, 60.0–89.0, and ≥ 90.0 mL/min/1.73 m^2^ were 11 (10.2%), 28 (25.9%), 48 (44.4%), and 21 (19.4%), respectively. Approximately, a half of the patients had serum albumin concentration of > 1.50 g/dL (n = 55 [50.9%]). Median level of proteinuria was 7.8 g/day or g/gCr (5.1, 10.7). Within 1 month of IST, most patients (n = 107 [99.1%]) received oral prednisolone (PSL) and intravenous methylprednisolone (mPSL) was administered in an approximately quarter of patients (n = 28 [25.9%]).

**Table 1:** Clinical characteristics of 108 adult patients with minimal change disease stratified by serum albumin concentration and estimated glomerular filtration rate.

	All	Serum albumin, g/dL		eGFR, mL/min/1.73 m^2^	
		≤ 1.50	> 1.50	< 60.0	≥ 60.0
Number	108	53	55	39	69
**Baseline characteristics at initiating IST**					
Age, years^†^	43 (30, 64)	37 (26, 60)	48 (33, 68)	55 (35, 74)	39 (27, 56)
18–39 years, n (%)^†^	49 (45.4)	29 (54.7)	20 (36.4)	12 (30.8)	37 (53.6)
40–64	32 (29.6)	13 (24.5)	19 (34.5)	12 (30.8)	20 (29.0)
65–81	27 (25.0)	11 (20.8)	16 (29.1)	15 (38.5)	12 (17.4)
Male, n (%)	66 (61.1)	35 (66.0)	31 (56.4)	26 (66.7)	40 (58.0)
Body mass index, kg/m^2^	24.1 ± 4.2	24.2 ± 4.1	23.9 ± 4.3	25.5 ± 4.6	23.3 ± 4.5
Systolic blood pressure, mmHg^†^	121 ± 16	120 ± 15	123 ± 16	122 ± 15	124 ± 18
Diastolic blood pressure, mmHg	73 ± 11	71 ± 11	74 ± 10	75 ± 11	72 ± 11
Serum creatinine, mg/dL^†^	0.87 (0.70, 1.24)	0.96 (0.71, 1.25)	0.84 (0.70, 1.22)	1.38 (1.18, 2.18)	0.72 (0.65, 0.87)
eGFR, mL/min/1.73 m^2^	67 ± 27	67 ± 25	67 ± 29	42 (24, 46)	81 (72, 93)
< 30.0 mL/min/1.73 m^2^	11 (10.2)	5 (9.4)	6 (10.9)	11 (28.2)	
30.0–59.9	28 (25.9)	15 (28.3)	13 (23.6)	28 (71.8)	
60.0–89.9	48 (44.4)	23 (43.4)	25 (45.5)		48 (69.6)
≥ 90.0	21 (19.4)	10 (18.9)	11 (20.0)		21 (30.4)
Serum albumin, g/dL	1.7 ± 0.6	1.20 (1.10, 1.40)	2.00 (1.80, 2.30)	1.64 ± 0.52	1.69 ± 0.58
≤ 1.00 g/dL, N (%)	12 (11.1)	12 (22.6)		3 (7.7)	9 (13.0)
1.01–1.50	41 (38.0)	41 (77.4)		17 (43.6)	24 (34.8)
1.51–2.00	28 (25.9)		28 (50.9)	10 (25.6)	18 (26.1)
> 2.00	27 (25.0)		27 (49.1)	9 (23.1)	18 (26.1)
Urinary protein, g/day or g/gCr^†^	7.8 (5.1, 10.7)	7.9 (5.3, 10.5)	7.8 (5.0, 10.8)	8.0 (5.0, 13.4)	7.7 (5.3, 9.9)
Dipstick hematuria, − or ±, N (%)^†^	48 (44.4)	25 (47.2)	23 (41.8)	9 (23.1)	39 (56.5)
1+	20 (18.5)	8 (15.1)	12 (21.8)	9 (23.1)	11 (15.9)
≥ 2+	40 (37.0)	20 (37.7)	20 (36.4)	21 (53.8)	19 (27.5)
RAS blockade, n (%)^†^	15 (13.9)	6 (11.3)	9 (16.4)	10 (25.6)	5 (7.2)
Intravenous albumin administration, n (%)*^†^	12 (11.1)	2 (3.7)	10 (18.2)	8 (20.5)	4 (5.8)
**Use of immunosuppressive drugs within 1 month of IST**					
Oral PSL, n (%)	107 (99.1)	53 (100.0)	54 (98.2)	39 (100.0)	68 (98.6)
Intravenous mPSL, n (%)	28 (25.9)	15 (28.3)	13 (23.6)	14 (35.9)	14 (20.3)
Cyclosporine, n (%)	12 (11.1)	4 (7.5)	8 (14.5)	5 (12.8)	7 (10.1)
Rituximab, n (%)	1 (0.9)	0 (0.0)	1 (1.8)	0 (0.0)	1 (1.4)
**Cumulative incidence of remission and relapse**					
Remission, n (%)	104 (96.3)	52 (98.1)	52 (94.5)	38 (97.4)	66 (95.7)
Remission within 6 months of IST, n (%)*	97 (89.8)	51 (96.2)	46 (83.6)	33 (84.6)	64 (92.8)
Relapse after remission, n (%)^‡^	42 (43.3)	24 (47.1)	18 (39.1)	15 (45.5)	27 (42.2)

Mean ± standard deviation; median (25%, 75%).

*eGFR* estimated glomerular filtration rate, *IST* immunosuppressive therapy, *mPSL* methylprednisolone, *PSL* prednisolone, *RAS* renin-angiotensin system.

*P < 0.05 between ≤ 1.50 and > 1.50 g/dL of serum albumin concentration for the t test, the Wilcoxson rank-sum test, the chi-square test, or the Fisher’s exact test, as appropriately.

^†^P < 0.05 between < 60.0 and ≥ 60.0 mL/min/1.73 m^2^ of eGFR for the unpaired t test, the Wilcoxson rank-sum test, the chi-square test, or the Fisher’s exact test, as appropriately.

^‡^Cumulative incidence of relapse in 97 patients with remission within 6 months of IST.

The clinical characteristics of 108 adult patients with MCD stratified by serum albumin levels were listed in Table S1, including 12 (11.1%), 41 (38.0%), 28 (25.9%), and 27 (25.0%) patients with serum albumin concentrations of ≤ 1.00, 1.01–1.50, 1.51–2.00, and > 2.00 g/dL, respectively. Age, urinary protein level, and intravenous albumin administration at initiating IST were significantly different among 4 groups of serum albumin concentration. After categorizing 108 patients into 2 groups of ≤ 1.50 (n = 53 [49.1%]) and > 1.50 g/dL (n = 55 [50.9%]) of serum albumin concentration, no significant difference was observed between these groups in baseline characteristics and use of immunosuppressive drugs within one month of IST, except intravenous albumin administration (Table 1). Table S2 shows the clinical characteristics stratified by estimated GFR (eGFR) groups, including 11 (10.2%), 28 (25.9%), 48 (44.4%), and 21 (19.4%) patients with eGFR < 30.0, 30.0–59.9, 60.0–89.9, and ≥ 90.0 mL/min/1.73 m^2^, respectively. Age, age category, body mass index (BMI), and systolic and diastolic blood pressure, serum creatinine concentration, dipstick hematuria, renin-angiotensin system (RAS) blockade at initiating IST were significantly different among the eGFR groups. Between the patients with eGFR ≥ 60 mL/min/1.73 m^2^ and those with eGFR < 60 mL/min/1.73 m^2^, age, age category, systolic blood pressure, serum creatinine concentration, urinary protein level, dipstick hematuria, RAS blockade, and intravenous albumin administration at initiating IST were significantly different (Table 1). The clinical characteristics stratified by urinary protein groups are listed in Table S3. Age, age category, BMI, serum creatinine concentration at initiating IST were significantly different among 4 groups of urinary protein, besides use of intravenous mPSL within 1 month of IST.

**Figure 1:**
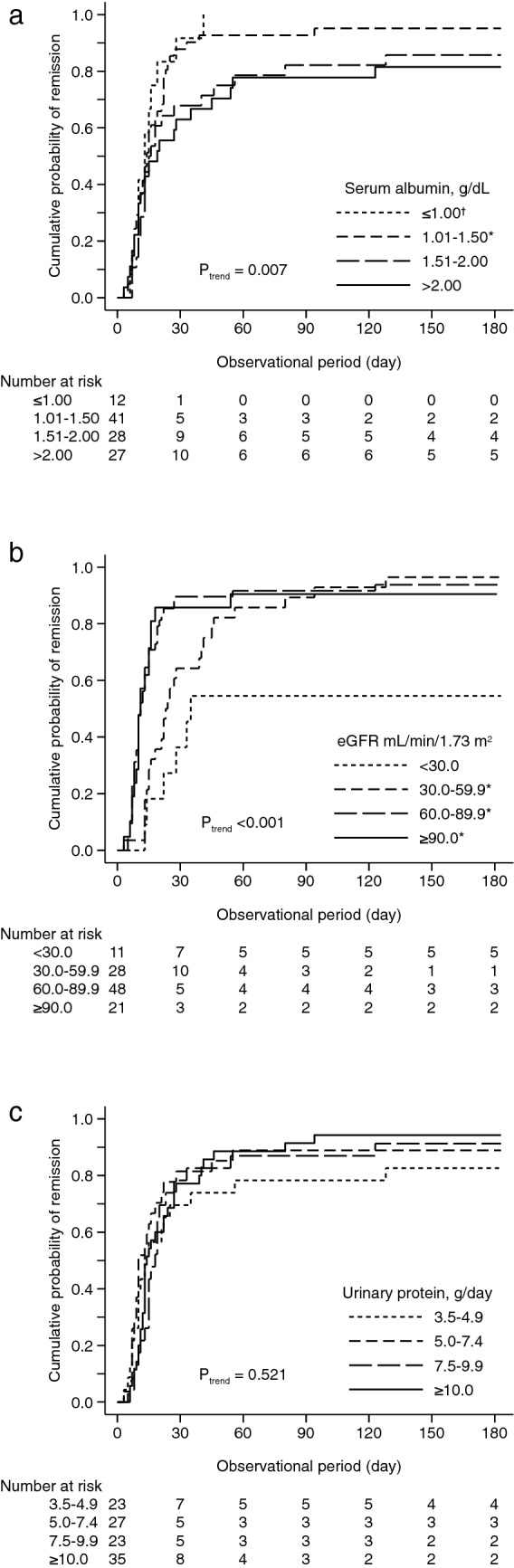
Cumulative probability of remission stratified by serum albumin concentration (**a**), eGFR level (**b**), and urinary protein level (**c**). *P < 0.05, vs. serum albumin concentration > 2.00 g/dL and eGFR < 30.0 mL/min/1.73 m^2^. ^†^P < 0.10, vs. serum albumin concentration > 2.00 g/dL.

During the median (interquartile range) observational period of 15 (10, 28) days, remission was observed in 12 (100.0%), 40 (97.6%), 28 (100.0%), and 24 (88.9%) patients with serum albumin levels of ≤ 1.00, 1.01–1.50, 1.51–2.00, and > 2.00 g/dL, respectively (Table S1). Patients with lower serum albumin concentrations were likely to achieve remission more rapidly (P_trend_ = 0.007) (Fig. 1a). Compared with patients with > 2.00 g/dL of serum albumin concentration, those with 1.01–1.50 g/dL had a significantly lower cumulative probability of remission (P = 0.046) and those with ≤ 1.00 g/dL had lower cumulative probability of remission at marginally significant level (P = 0.060). In patients with eGFR of < 30.0, 30.0–59.9, 60.0–89.9, and ≥ 90.0 mL/min/1.73 m^2^, 10 (90.9%), 28 (100.0%), 46 (95.8%), and 20 (95.2%) patients achieved remission, respectively (Table S2). Patients with a higher eGFR were more likely to achieve remission more rapidly (P_trend_ < 0.001) (Fig. 1b). Compared with patients with eGFR < 30.0 mL/min/1.73 m^2^, those with eGFR ≥ 30.0 mL/min/1.73 m^2^ had a significantly higher cumulative probability of remission. In contrast, no significant difference was observed in the cumulative incidence of remission among the four groups of urinary protein levels (Fig. 1c). Unadjusted Cox proportional hazards (CPH) models showed that younger age, lower systolic blood pressure, lower serum albumin concentration, and higher eGFR level were significantly associated with remission (Table 2). A multivariable-adjusted model identified serum albumin (per 1.0 g/dL, adjusted hazard ratio [HR] 0.57 [95% confidence interval 0.37, 0.87]) and eGFR (per 30 mL/min/1.73 m^2^, 1.43 [1.08, 1.90]) as significant predictors of remission (Table 2).

**Table 2:** Predictors of remission and relapse.

	Remission (n = 108)		Relapse after remission (n = 97)^†^	
	Unadjusted HR (95% CI)	Adjusted HR (95% CI)^‡^	Unadjusted HR (95% CI)	Adjusted HR (95% CI)^‡^
Age, 18–39 years	1.00 (reference)	1.00 (reference)	1.00 (reference)	1.00 (reference)
40–64	0.98 (0.62, 1.53)	1.39 (0.83, 2.32)	0.75 (0.38, 1.49)	0.90 (0.40, 2.04)
65–81	0.54 (0.33, 0.89)*	0.73 (0.40, 1.33)	0.82 (0.35, 1.93)	1.21 (0.44, 3.39)
Men	1.16 (0.78, 1.73)	1.42 (0.89, 2.29)	0.77 (0.42, 1.43)	0.88 (0.44, 1.76)
Body mass index, per 1.0 kg/m^2^	0.96 (0.92, 1.01)	0.95 (0.89, 1.01)	1.00 (0.92, 1.09)	1.04 (0.93, 1.16)
Systolic blood pressure, per 10 mmHg	0.89 (0.80, 0.99)*	1.00 (0.85, 1.17)	0.88 (0.70, 1.10)	0.87 (0.64, 1.18)
Serum albumin, per 1.0 g/dL	0.65 (0.44, 0.95)*	0.57 (0.37, 0.87)*	0.73 (0.43, 1.23)	0.93 (0.52, 1.66)
eGFR, per 30 mL/min/1.73 m^2^	1.33 (1.10, 1.62)*	1.43 (1.08, 1.90)*	0.93 (0.65, 1.32)	0.91 (0.58, 1.44)
UP, per 1.0 log g/day or log g/gCr	1.07 (0.76, 1.52)	1.25 (0.79, 2.00)	1.30 (0.72, 2.33)	1.05 (0.99, 1.13)
Dipstick hematuria, − or ±	1.00 (reference)	1.00 (reference)	1.00 (reference)	1.00 (reference)
1+	0.86 (0.50, 1.47)	1.31 (0.71, 2.41)	0.82 (0.34, 1.96)	0.64 (0.24, 1.70)
≥ 2+	0.72 (0.47, 1.10)	0.92 (0.55, 1.53)	1.40 (0.72, 2.72)	1.57 (0.69, 3.55)
Intravenous albumin administration	0.67 (0.37, 1.23)	0.97 (0.44, 2.15)	0.47 (0.11, 1.96)	0.15 (0.02, 1.25)
Intravenous mPSL within 1 month of IST	0.66 (0.42, 1.03)	0.68 (0.41, 1.11)	0.96 (0.47, 1.94)	0.86 (0.40, 1.85)
Cyclosporine within 1 month of IST	0.58 (0.31, 1.09)	0.65 (0.32, 1.33)	0.64 (0.23, 1.80)	0.87 (0.28, 2.65)

*CI* confidence interval, *eGFR* estimated glomerular filtration rate, *HR* hazard ratio, *mPSL* methylprednisolone, *UP* urinary protein.

*P < 0.05.

^†^Including 97 patients with remission of proteinuria within 6 months of IST.

^‡^Adjusted for all variables listed in the table.

**Figure 2:**
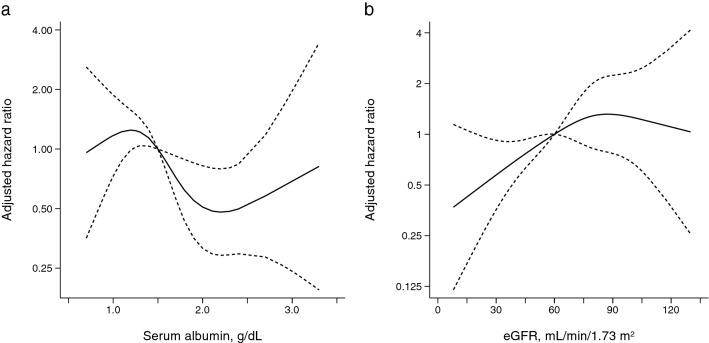
Restricted cubic spline curve for the association of serum albumin (**a**) and eGFR (**b**) with remission, adjusted for age (18–40, 41–64, and ≥ 65 years), sex, body mass index (kg/m^2^), systolic blood pressure (mmHg), urinary protein (g/day or g/gCr), eGFR (mL/min/1.73 m^2^, if serum albumin), serum albumin concentration (g/dL, if eGFR), dipstick hematuria (− or ± , 1+, and ≥ 2+), and intravenous albumin administration at initiating IST; and use of intravenous methylprednisolone and cyclosporine within one month after initiating immunosuppressive therapy.

To clarify the dose-dependent association of serum albumin concentration and eGFR with remission, the unadjusted and adjusted HR of each group of serum albumin and eGFR was calculated. Compared with patients with serum albumin concentration of > 2.00 g/dL, those with serum albumin concentration of ≤ 1.00 and 1.01–1.50 g/dL had significantly higher unadjusted and adjusted HR, and their HRs were very comparable (adjusted HRs of serum albumin concentration of ≤ 1.00, 1.01–1.50, 1.51–2.00, and > 2.00 g/dL: 2.47 [1.14, 5.34], 2.32 [1.31, 4.14], 1.51 [0.83, 2.73], and 1.00 [reference], respectively) (Table 3). A multivariable-adjusted restricted cubic spline model confirmed the non-linear association between serum albumin concentration and remission (Fig. 2a). A similar non-linear association was observed between eGFR and remission. Compared with patients with eGFR of < 30.0 mL/min/1.73 m^2^, those with eGFR of 60.0–89.9 and ≥ 90.0 mL/min/1.73 m^2^ were significantly associated with remission at the similar level (adjusted HRs of eGFR of < 30.0, 30.0–59.9, 60.0–89.9, and ≥ 90.0 mL/min/1.73 m^2^: 1.00 [reference], 1.21 [0.54, 2.70], 2.59 [1.18, 5.70], and 2.73 [1.09, 6.84], respectively) (Table 3). The non-linear association between eGFR and remission was verified in a multivariable-adjusted restricted cubic spline model (Fig. 2b). According to the non-linear association of serum albumin concentration and eGFR, we categorized the patients into four groups based on eGFR (< 60.0 vs. ≥ 60.0 mL/min/1.73 m^2^) and serum albumin concentration (> 1.50 vs. ≤ 1.50 g/dL) and calculated their HRs. Compared with patients with eGFR ≥ 60.0 mL/min/1.73 m^2^ and serum albumin concentration > 1.50 g/dL, those with eGFR < 60.0 mL/min/1.73 m^2^ and serum concentration > 1.50 g/dL achieved remission significantly more slowly (0.48 [0.23, 1.00]), whereas those with eGFR ≥ 60.0 mL/min/1.73 m^2^ and serum concentration ≤ 1.50 g/dL did significantly more rapidly (2.20 [1.28, 3.81]) (Table 3).

**Table 3:** Serum albumin, eGFR, and the incidence of remission.

Category	N	Remission N (%)	Unadjusted HR (95% CI)	Adjusted HR (95% CI)^†^
**Serum albumin**				
≤ 1.00 g/dL	12	12 (100.0)	2.18 (1.07, 4.46)*	2.47 (1.14, 5.34)*
1.01–1.50	41	40 (97.6)	1.79 (1.06, 3.01)*	2.32 (1.31, 4.14)*
1.51–2.00	28	28 (100.0)	1.27 (0.73, 2.20)	1.51 (0.83, 2.73)
> 2.00	27	24 (88.9)	1.00 (reference)	1.00 (reference)
**eGFR**				
< 30.0 mL/min/1.73 m^2^	11	10 (90.9)	1.00 (reference)	1.00 (reference)
30.0–59.9	28	28 (100.0)	1.69 (0.81, 3.52)	1.21 (0.54, 2.70)
60.0–89.9	48	46 (95.8)	2.90 (1.45, 5.81)*	2.59 (1.18, 5.70)
≥ 90.0	21	20 (95.2)	2.81 (1.30, 6.05)*	2.73 (1.09, 6.84)
**eGFR and serum albumin**				
< 60.0 mL/min/1.73 m^2^ and > 1.50 g/dL	19	18 (94.7)	0.50 (0.28, 0.89)*	0.48 (0.23, 1.00)*
< 60.0 and ≤ 1.50	20	20 (100.0)	0.82 (0.47, 1.43)	0.85 (0.45, 1.59)
≥ 60.0 and > 1.50	36	34 (94.4)	1.00 (reference)	1.00 (reference)
≥ 60.0 and ≤ 1.50	33	32 (97.0)	1.84 (1.13, 3.02)*	2.20 (1.28, 3.81)*

*CI* confidence interval, *eGFR* estimated glomerular filtration rate, *IRR* incidence rate ratio.

*P < 0.05.

^†^Adjusted for age (18–40, 41–64, and ≥ 65 years), sex, body mass index (kg/m^2^), systolic blood pressure (mmHg), serum albumin (g/dL, if eGFR), eGFR (mL/min/1.73 m^2^, if serum albumin), urinary protein (log g/day or log g/gCr), dipstick hematuria (− or ± , 1+, and ≥ 2+), use of intravenous albumin before immunosuppressive therapy, and use of intravenous methylprednisolone and cyclosporine within 1 month after initiating immunosuppressive therapy.

Predictors of relapse of proteinuria were assessed in 97 patients with remission within 6 months of IST. During the median (interquartile range) observational period of 2.2 [0.9, 4.7] years, relapse was observed in 3 (25.0%), 21 (53.8%), 11 (45.8%), and 7 (31.8%) patients with serum albumin concentration of ≤ 1.00, 1.01–1.50, 1.51–2.00, and > 2.00 g/dL, respectively (Table S1). No significant difference was observed in the cumulative probability of relapse among the four groups of serum albumin concentrations (P_trend_ = 0.407). Regarding the four eGFR groups, 3 (50.0%), 12 (44.4%), 18 (40.0%), and 9 (47.4%) patients with eGFR of < 30.0, 30.0–59.9, 60.0–89.9, and ≥ 90.0 mL/min/1.73 m^2^ relapsed after remission, respectively (Table S2). The cumulative probability of relapse was comparable among these eGFR groups (P_trend_ = 0.633). Unadjusted and adjusted CPH models showed that no variable was associated with relapse (Table 2).

## Discussion

The present study clarified that serum albumin and eGFR were associated with remission of proteinuria in a non-linear fashion in 108 adult patients with MCD, whereas they were not associated with relapse of proteinuria. Patients with lower serum albumin concentrations, especially ≤ 1.5 g/dL, were likely to achieve remission more rapidly. Lower eGFR, especially < 60 mL/min/1.73 m^2^, was associated with slower remission. An advantage of the present study was the detailed assessment of the multivariable-adjusted non-linear association of serum albumin and eGFR with remission, providing clinically useful information to identify the patients who are resistant to IST, namely, those with serum albumin concentration > 1.5 g/dL or eGFR < 60 mL/min/1.73 m^2^.

Conflicting associations between serum albumin concentration and remission of proteinuria in patients with MCD have been reported in some retrospective cohort studies. A retrospective single-center cohort study in the UK, including 51 adult patients with MCD at a single hospital, reported that the time to remission was positively correlated with serum albumin concentration^10^, compatible with the results of the present study. A Japanese retrospective single-center cohort study, including 53 adult patients with MCD, verified the inverse association between serum albumin concentration and remission, even after adjusting for potential clinical confounding factors^15^. In contrast, two cohort studies reported no significant association between serum albumin concentration and remission. A retrospective single-center cohort study in the UK, including 52 adult patients with MCD, showed that serum albumin concentration was not associated with remission in an unadjusted CPH model^21^. Another Japanese retrospective multicenter cohort study, the STudy of Outcomes and Practice patterns of Minimal Change Disease (STOP-MCD), including 142 adult patients with MCD in five hospitals showed no significant association between serum albumin concentration and remission in a multivariable-adjusted CPH model^13^. In the STOP-MCD study, high prevalence of intravenous albumin administration (62.0% in the STOP-MCD study vs. 11.1% in the JNSCS) might blunted the association between serum albumin concentration and remission. The present multicenter prospective cohort study with higher external validity than previous studies, including 108 adult patients with MCD in 40 hospitals in Japan, showed that serum albumin concentration was inversely associated with remission.

Previous studies have reported contradictory impacts of kidney function on remission of proteinuria in patients with MCD. A retrospective single-center cohort study, including 52 patients with MCD in UK, reported that eGFR was not associated with remission in an unadjusted CPH model^21^. Inclusion of suspected secondary MCD (11.5%) might potentially dilute the association between eGFR and remission. In contrast, a Japanese single-center retrospective cohort study suggested that higher serum creatinine level was associated with slower remission in 53 adult patients with MCD^11^. Another Japanese retrospective multicenter cohort study, the STOP-MCD study, including 142 adult patients with MCD in 5 hospitals, confirmed the inverse association between serum creatinine level and remission using a multivariable-adjusted CPH model^13^. The present study ascertained that patients with lower kidney function, especially eGFR < 60 mL/min/1.73 m^2^, achieved remission more slowly. The sample size was comparable to the previous largest Japanese study^13^ and a large number of participating hospitals provided evidence with high external validity.

The present study has several limitations. First, the association between low eGFR and slower remission might be confounded by AKI. Of 716 patients with MCD included in 13 reports, AKI was commonly observed in 235 (33.3%) patients^22^. A Taiwanese retrospective cohort study of MCD reported that 23 patients with no AKI and creatinine clearance of 88.3 ± 23.6 mL/min had a significantly higher cumulative probability of remission than 20 patients with AKI and creatinine clearance of 31.6 ± 19.2 mL/min^12^. Another Japanese cohort study, including 53 adult patients with MCD, clarified a dose-dependent association between AKI stage of the Kidney Disease Improving Global Outcomes (KDIGO) criteria and remission, using CPH model adjusting for clinically relevant factors except for eGFR^15^. In the present study, patients with AKI and, therefore, lower eGFR might achieve remission more slowly than those with no AKI and higher eGFR. The limited number of eGFR measurements available in the present study hindered the identification of the incidence of AKI during the clinical course of each patient. The clinical impact of AKI on the association between eGFR and remission should be assessed in future studies. Second, details of IST, including the time between the onset of symptomatic edema and IST, the initial dose of PSL, and the total duration of prednisolone use, were not available in the present study. Because of the observational nature of the present study, the lack of IST protocol potentially led to biased results. Thus, the associations of use of intravenous mPSL and cyclosporine with remission and relapse in the present study should be interpreted with great caution. The JNSCS is planning to retrieve all laboratory and drug data of each patient during the observational period, which will enable statistical methods for modeling time-updated exposure to IST^23^ to estimate precise effectiveness of IST in a real-world setting.

In conclusion, this multicenter prospective cohort study clarified that higher serum albumin concentrations and lower eGFR levels were independently associated with a lower cumulative probability remission in adult patients with MCD. The findings of the present study provide a simple risk stratification system for remission in adult patients with MCD, which should be verified in different cohorts.

## Methods

**Figure 3:**
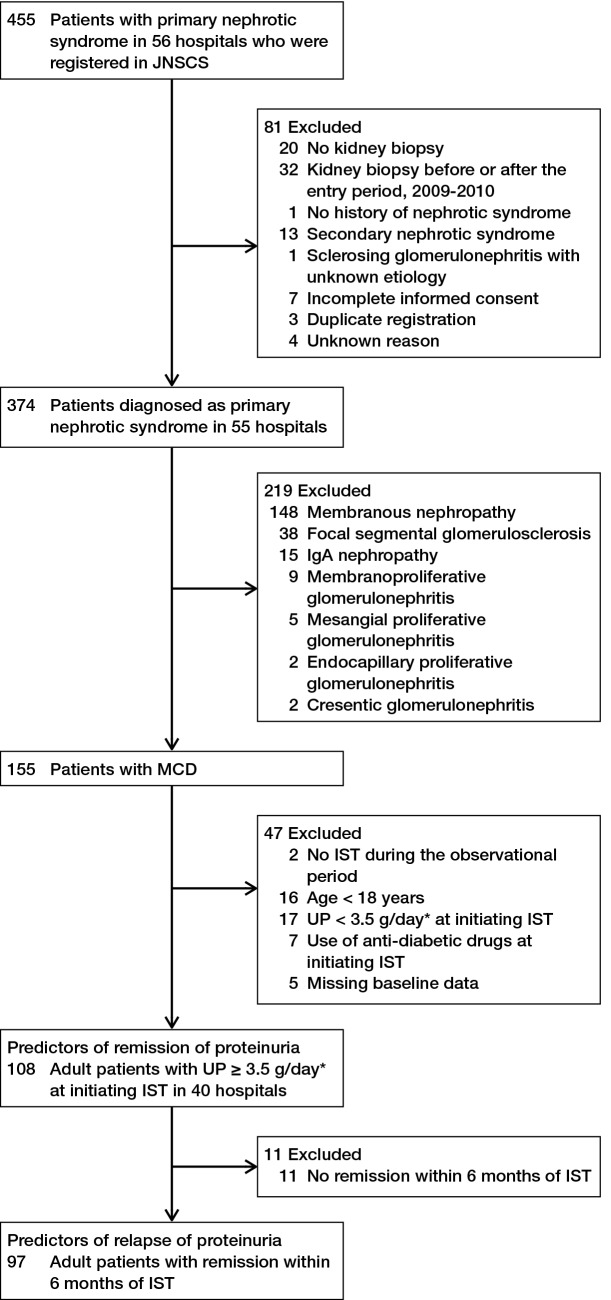
Flow diagram of inclusion and exclusion of study participants.

The JNSCS, a 5-year multicenter prospective cohort study of primary nephrotic syndrome, aimed to clarify the incidence rates of major clinical outcomes and assess the effectiveness of IST in Japan^16,17^. Of 455 nephrotic patients who were diagnosed with primary nephrotic syndrome between January 2009 and December 2010 in 56 hospitals and registered in the JNSCS, 81 patients including those with no kidney biopsy (n = 20), kidney biopsy before or the entry period (n = 32), no history of nephrotic syndrome (n = 1), diagnosis of secondary nephrotic syndrome (n = 13), sclerosing glomerulonephritis with unknown etiology (n = 1), incomplete informed consent (n = 7), duplicate registration (n = 3), and unknown reasons (n = 4) were excluded (Fig. 3). Finally, the JNSCS enrolled 374 patients with primary nephrotic syndrome in 55 hospitals, including those with MCD (n = 155), MN (n = 148), FSGS (n = 38), IgA nephropathy (n = 15), membranoproliferative glomerulonephritis (n = 9), mesangial proliferative glomerulonephritis (n = 5), endocapillary proliferative glomerulonephritis (n = 2), and crescentic glomerulonephritis (n = 2). Of 155 patients with MCD, 108 adult patients aged 18 years or older with urinary protein ≥ 3.5 g/day at initiating IST in 40 hospitals were included to identify the predictors of remission of proteinuria after initiating IST, after excluding two patients without IST during the observational period, 16 patients aged < 18 years, 17 patients with urinary protein < 3.5 g/day at initiating IST, 7 patients with use of anti-diabetic drugs at initiating IST, and 5 patients with missing baseline data at initiating IST. To identify predictors of relapse of proteinuria, 97 patients with remission within 6 months of IST were included after excluding 11 patients with no remission within 6 months of IST, because 93.3% of patients with remission during the entire observational period achieve remission within 6 months of IST.

The study protocol of the JNSCS was approved by the ethics committee of Osaka University Hospital (approval number 17035-4) and the Institutional Review Board of each participating hospital. All procedures performed in the JNSCS involving human participants were in accordance with the ethical standards of the research committee of the institute at which the studies were conducted and with the 1964 Helsinki declaration and its later amendments or comparable ethical standards. Written informed consent was obtained from all participants and the legal representatives of the participants under 20 years of age in 54 hospitals. A single hospital used an opt-out approach to provide informed consent, according to the Japanese Ethical Guidelines for Medical and Health Research Involving Human Subjects.

## Measurements

Baseline characteristics at initiation of IST included age, sex, BMI, systolic and diastolic blood pressure, serum creatinine and albumin concentration, eGFR, 24 h urinary protein (or urinary protein-to-creatinine ratio if 24 h urinary protein was missing), and RAS blockade, including use of angiotensinogen converting enzyme inhibitors and angiotensin II receptor blockers, and intravenous albumin administration. The Japanese equation was used to calculate eGFR: eGFR = 194 × age (year)^−0.287^ × serum creatinine (mg/dL)^−0.094^ × 0.739 (if female)^24^. Data pertaining to the use of immunosuppressive drugs within 1 month of IST were also collected, including oral prednisolone, intravenous mPSL, cyclosporine, tacrolimus, cyclophosphamide, mycophenolate mofetil, mizoribine, and rituximab.

The outcome of interest was (i) remission of proteinuria defined as 24-h urinary protein < 0.3 g/day or urinary protein-to-creatinine ratio of < 0.3 g/gCr, and (ii) relapse of proteinuria defined as 24-h urinary protein ≥ 1.0 g/day, urinary protein-to-creatinine ratio ≥ 1.0 g/gCr, and/or dipstick urinary protein ≥ 2+ continued two or more times. The observational period to identify the predictors of remission was defined as the period from the initiation of IST to (i) the incidence of remission, (ii) the end of the 5-year study period of the JNSCS, or (iii) loss to follow-up, whichever came first. To identify the predictors of relapse, the observational period was defined as the period from the incidence of remission to (i) the incidence of relapse, (ii) the end of the 5-year study period of the JNSCS, or (iii) loss to follow-up, whichever came first.

### Statistics

After categorizing serum albumin concentration into four groups of ≤ 1.00, 1.01–1.50, 1.51–2.00, and > 2.00 g/day L; eGFR into 4 groups of < 30.0, 30.0–59.9, 60.0–89.9, and ≥ 90.0 mL/min/1.72 m^2^; and urinary protein into 4 groups of 3.5–4.9, 5.0–7.4, 7.5–9.9, ≥ 10.0 g/day or g/gCr, baseline characteristics, use of immunosuppressive drugs within 1 month of IST, and the cumulative incidence of remission and relapse were compared among these 4 groups using analysis of variance, the Kruskal–Wallis test, the chi-square test, and the Fisher’s exact test, as appropriate. We also compared these clinical characteristics after categorizing the patients into two categories of serum concentration of ≤ 1.50 and > 1.50 g/dL and eGFR of < 60.0 and ≥ 60.0 mL/min/1.73 m^2^, using the unpaired t-test, the Wilcoxon rank-sum test, or the chi-square test, as appropriate.

Cumulative probabilities of remission in the four groups of serum albumin concentration, eGFR level, and urinary protein level were calculated using the Kaplan–Meier method and compared using log-rank test for trend. To identify predictors of remission and relapse, we used unadjusted and multivariable-adjusted CPH models, including age (18–39, 40–64, and ≥ 65 year), sex, BMI (kg/m^2^), systolic blood pressure (mmHg), serum albumin (g/dL), eGFR (mL/min/1.73 m^2^), urinary protein (log g/day or log g/gCr), dipstick hematuria (− or ± , 1+, and ≥ 2+), intravenous albumin administration, and use of intravenous mPSL and cyclosporine within 1 month of IST as covariates. Because of its skewed distribution, urinary protein was included in CPH models after logarithmic transformation.

To clarify a dose-dependent association of serum albumin and eGFR with remission, we used restricted cubic spline functions using 4 knots placed at 5th, 35th, 65th, and 95th percentiles^25^ of serum albumin (0.85, 1.40, 1.80, and 2.70 g/dL, respectively) and eGFR (16, 59, 80, and 107 mL/min/1.73 m^2^, respectively). The cutoff values between the second and third groups, namely serum albumin of 1.5 g/dL and eGFR of 60 mL/min/1.73 m^2^, were used as the reference, which were very close to the median values of these variables (1.59 g/dL and 70 mL/min/1.73 m^2^, respectively).

Continuous variables were expressed as the mean ± standard deviation or median and interquartile range, as appropriate, and categorical variables were expressed as numbers and proportions. Statistical significance was set at P < 0.05. Statistical analyses were performed using Stata, version 17.0 (Stata Corp, www.stata.com).

## Supplementary Information

Supplementary Tables.

## Data Availability

The data that support the findings of this study are available from the corresponding author upon reasonable request and with permission of the Steering Committee for the JNSCS.
