# Supplementary material for: Predictors of early remission of proteinuria in adult patients with minimal change disease: a retrospective cohort study
Source: Sci Rep. 2022 Jun 13;12:9782. doi: 10.1038/s41598-022-13067-7 (PMC9192725; doi:10.1038/s41598-022-13067-7)
Supplement: Supplementary file 1 — Supplementary Tables. [file 41598_2022_13067_MOESM1_ESM.pdf]

Supplementary tables

Table S1. Clinical characteristics of 108 adult patients with minimal change disease stratified by serum albumin concentration of ≤1.00, 1.01–1.50, 1.51–2.00, and >2.00 g/dL.

Table S2. Clinical characteristics of 108 adult patients with minimal change disease stratified by eGFR level of <30.0, 30.0–59.9, 60.0–89.9, and ≥90.0 mL/min/1.73 m^2

Table S3. Clinical characteristics of 108 adult patients with minimal change disease stratified by urinary protein level of 3.5–4.9, 5.0–7.4, 7.5–9.9, and ≥10.0 g/day or g/gCr.

Table S1. Clinical characteristics of 108 adult patients with minimal change disease stratified by serum albumin concentration.

Serum albumin, g/dL		All	≤1.00	1.01–1.50	1.51–2.00	>2.00
		1.7±0.6	0.9 (0.8, 0.9)	1.4 (1.2, 1.5)	1.8 (1.7, 2.0)	2.3 (2.2, 2.7)
Number		108	12	41	28	27
**Baseline characteristics at initiating IST**						
Age, years*		43 (30, 64)	30 (21, 40)	42 (26, 65)	48 (32, 73)	44 (37, 58)
18–39 years, n (%)		49 (45.4)	9 (75.0)	20 (48.8)	11 (39.3)	9 (33.3)
40–64		32 (29.6)	3 (25.0)	10 (24.4)	7 (25.0)	12 (44.4)
65–81		27 (25.0)	0 (0.0)	11 (26.8)	10 (35.7)	6 (22.2)
Male, n (%)		66 (61.1)	7 (58.3)	28 (68.3)	15 (53.6)	16 (59.3)
Body mass index, kg/m^2		24.1±4.2	26.3±3.7	23.6±4.1	23.9±4.3	23.9±4.4
Systolic blood pressure, mmHg		121±16	119±15	121±16	122±15	124±18
Diastolic blood pressure, mmHg		73±11	69±13	72±11	73±10	76±10
Serum creatinine, mg/dL		0.87 (0.70, 1.24)	0.97 (0.70, 1.23)	0.95 (0.71, 1.25)	0.87 (0.70, 1.16)	0.83 (0.66, 1.50)
eGFR, mL/min/1.73 m^2		67±27	71±21	66±27	66±25	67±33
<30 mL/min/1.73 m^2		11 (10.2)	0 (0.0)	5 (12.2)	1 (3.6)	5 (18.5)
30–59		28 (25.9)	3 (25.0)	12 (29.3)	9 (32.1)	4 (14.8)
60–89		48 (44.4)	6 (50.0)	17 (41.5)	13 (46.4)	12 (44.4)
≥90		21 (19.4)	3 (25.0)	7 (17.1)	5 (17.9)	6 (22.2)
Urinary protein, g/day or g/gCr*		7.8 (5.1, 10.7)	10.1 (8.0, 13.5)	7.0 (5.1, 10.0)	8.6 (5.6, 12.9)	7.1 (4.2, 9.5)
Dipstick hematuria	- or ±, N (%)	48 (44.4)	4 (33.3)	21 (51.2)	10 (35.7)	13 (48.1)
	1+	20 (18.5)	3 (25.0)	5 (12.2)	7 (25.0)	5 (18.5)
	≥2+	40 (37.0)	5 (41.7)	15 (36.6)	11 (39.3)	9 (33.3)
RAS blockade, n (%)		15 (13.9)	0 (0.0)	6 (14.6)	5 (17.9)	4 (14.8)
Intravenous albumin administration, n (%)*		12 (11.1)	1 (8.3)	1 (2.4)	7 (25.0)	3 (11.1)
**Use of immunosuppressive drugs within 1 month of IST**						
Oral PSL, n (%)		107 (99.1)	12 (100.0)	41 (100.0)	27 (96.4)	27 (100.0)
Intravenous mPSL, n (%)		28 (25.9)	4 (33.3)	11 (26.8)	7 (25.0)	6 (22.2)
Cyclosporine, n (%)		12 (11.1)	1 (8.3)	3 (7.3)	3 (10.7)	5 (18.5)

Rituximab, n (%)	1 (0.9)	0 (0.0)	0 (0.0)	1 (3.6)	0 (0.0)
**Cumulative incidence of remission and relapse**					
Remission, n (%)	104 (96.3)	12 (100.0)	40 (97.6)	28 (100.0)	23 (88.9)
Remission within 6 months of IST, n (%)	97 (89.8)	12 (100.0)	39 (95.1)	24 (85.7)	22 (81.5)
Relapse after remission, n (%)†	42 (43.3)	3 (25.0)	21 (53.8)	11 (45.8)	7 (31.8)

Mean±standard deviation; median (25%, 75%)

eGFR, estimated glomerular filtration rate; IST, immunosuppressive therapy; mPSL, methylprednisolone; PSL, prednisolone; RAS, renin-angiotensin system

*P <0.05 for analysis of variance, the Kruskal-Wallis test, the chi-square test, or the Fisher's exact test, as appropriately.

†Incidence of relapse in 97 patients with remission within 6 months of IST.

Table S2. Clinical characteristics of 108 adult patients with minimal change disease stratified by eGFR level.

eGFR, mL/min/1.73 m^2	All<br>67±27	<30.0<br>16 (14–24)	30.0–59.9<br>44 (38–52)	60.0–89.9<br>77 (67–82)	≥90.0<br>101 (94–106)
Number	108	11	28	48	21
**Baseline characteristics at initiating IST**					
Age, years*	43 (30, 64)	51 (20–75)	59 (37–74)	46 (33–61)	26 (21–36)
18–39 years, n (%)*	49 (45.4)	4 (36.4)	8 (28.6)	19 (39.6)	18 (85.7)
40–64	32 (29.6)	3 (27.3)	9 (32.1)	18 (37.5)	2 (9.5)
65–81	27 (25.0)	4 (36.4)	11 (39.3)	11 (22.9)	1 (4.8)
Male, n (%)	66 (61.1)	8 (72.7)	18 (64.3)	28 (58.3)	12 (57.1)
Body mass index, kg/m^2*	24.1±4.2	26.2±3.8	25.2±4.9	23.9±3.9	21.9±3.2
Systolic blood pressure, mmHg*	121±16	133±13	125±18	121±15	112±10
Diastolic blood pressure, mmHg*	73±11	76±10	75±11	73±11	68±9
Serum creatinine, mg/dL*	0.87 (0.70, 1.24)	2.91 (2.23, 4.14)	1.24 (1.09–1.39)	0.74 (0.66–0.97)	0.70 (0.61–0.78)
Serum albumin, g/dL	1.67±0.56	1.88±0.60	1.54±0.46	1.67±0.55	1.73±0.64
≤1.00 g/dL, N (%)	12 (11.1)	0 (0.0)	3 (10.7)	6 (12.5)	3 (14.3)
1.01–1.50	41 (38.0)	5 (45.5)	12 (42.9)	17 (35.4)	7 (33.3)
1.51–2.00	28 (25.9)	1 (9.1)	9 (32.1)	13 (27.1)	5 (23.8)
>2.00	27 (25.0)	5 (45.5)	4 (14.3)	12 (25.0)	6 (28.6)
Urinary protein, g/day or g/gCr	7.8 (5.1, 10.7)	8.0 (4.8–12.4)	9.1 (5.0–13.4)	7.6 (5.3–10.2)	7.8 (5.4–9.5)
Dipstick hematuria - or ±, N (%)*	48 (44.4)	3 (27.3)	6 (21.4)	26 (54.2)	13 (61.9)
1+	20 (18.5)	1 (9.1)	8 (28.6)	7 (14.6)	4 (19.0)
≥2+	40 (37.0)	7 (63.6)	14 (50.0)	15 (31.3)	4 (19.0)
RAS blockade, n (%)*	15 (13.9)	3 (27.3)	7 (25.0)	5 (10.4)	0 (0.0)
Intravenous albumin administration, n (%)	12 (11.1)	2 (18.2)	6 (21.4)	4 (8.3)	0 (0.0)
**Use of immunosuppressive drugs within 1 month of IST**					
Oral PSL, n (%)	107 (99.1)	11 (100.0)	28 (100.0)	47 (97.9)	21 (100.0)
Intravenous mPSL, n (%)	28 (25.9)	5 (45.5)	9 (32.1)	8 (16.7)	6 (28.6)
Cyclosporine, n (%)	12 (11.1)	2 (18.2)	3 (10.7)	5 (10.4)	2 (9.5)

Rituximab, n (%)	1 (0.9)	0 (0.0)	1 (3.6)	0 (0.0)	0 (0.0)
**Cumulative incidence of remission and relapse**					
Remission, n (%)	104 (96.3)	10 (90.9)	28 (100.0)	46 (95.8)	20 (95.2)
Remission within 6 months of IST, n (%)*	97 (89.8)	6 (54.5)	27 (96.4)	45 (93.8)	19 (90.5)
Relapse after remission, n (%)†	42 (43.3)	3 (50.0)	12 (44.4)	18 (40.0)	9 (47.4)

Mean±standard deviation; median (25%, 75%)

eGFR, estimated glomerular filtration rate; IST, immunosuppressive therapy; mPSL, methylprednisolone; PSL, prednisolone; RAS, renin-angiotensin system

*P <0.05 for analysis of variance, the Kruskal-Wallis test, the chi-square test, or the Fisher's exact test, as appropriately.

†Incidence of relapse in 97 patients with remission within 6 months of IST.

Table S3. Clinical characteristics of 108 adult patients with minimal change disease stratified by urinary protein level.

Urinary protein, g/day or g/gCr	All<br>7.8 (5.1, 10.7)	3.5–4.9<br>4.1 (3.7, 4.6)	5.0–7.4<br>6.0 (5.3, 6.8)	7.5–9.9<br>8.2 (7.9, 8.9)	≥10.0<br>12.4 (10.7, 15.6)
Number	108	23	27	23	35
**Baseline characteristics at initiating IST**					
Age, years*	43 (30, 64)	52 (36, 73)	49 (36, 67)	35 (25, 50)	38 (26, 58)
18–39 years, n (%)*	49 (45.4)	7 (30.4)	9 (33.3)	14 (60.9)	19 (54.3)
40–64	32 (29.6)	5 (21.7)	9 (33.3)	7 (30.4)	11 (31.4)
65–81	27 (25.0)	11 (47.8)	9 (33.3)	2 (8.7)	5 (14.3)
Male, n (%)	66 (61.1)	15 (65.2)	14 (51.9)	15 (65.2)	22 (62.9)
Body mass index, kg/m^{2}*	24.1±4.2	22.3±3.6	23.9±3.4	23.8±3.4	25.5±5.1
Systolic blood pressure, mmHg	121±16	119±15	123±15	118±13	123±19
Diastolic blood pressure, mmHg	73±11	70±9	73±12	75±10	74±11
Serum creatinine, mg/dL*	0.87 (0.70, 1.24)	0.84 (0.70, 1.20)	0.74 (0.65, 1.18)	0.80 (0.70, 1.06)	1.02 (0.87, 1.76)
eGFR, mL/min/1.73 m^{2}	67±27	66±28	70±21	76±30	58±27
<30 mL/min/1.73 m^{2}	11 (10.2)	3 (13.0)	1 (3.7)	3 (13.0)	4 (11.4)
30–59	28 (25.9)	7 (30.4)	6 (22.2)	1 (4.3)	14 (40.0)
60–89	48 (44.4)	8 (34.8)	15 (55.6)	12 (52.2)	13 (37.1)
≥90	21 (19.4)	5 (21.7)	5 (18.5)	7 (30.4)	4 (11.4)
Serum albumin, g/dL	1.67±0.56	1.82±0.54	1.69±0.57	1.64±0.66	1.57±0.47
≤1.00 g/dL, N (%)	12 (11.1)	0 (0.0)	2 (7.4)	4 (17.4)	6 (17.1)
1.01–1.50	41 (38.0)	9 (39.1)	13 (48.1)	8 (34.8)	11 (31.4)
1.51–2.00	28 (25.9)	6 (26.1)	5 (18.5)	4 (17.4)	13 (37.1)
>2.00	27 (25.0)	8 (34.8)	7 (25.9)	7 (30.4)	5 (14.3)
Dipstick hematuria - or ±, N (%)	48 (44.4)	12 (52.2)	13 (48.1)	10 (43.5)	13 (37.1)
1+	20 (18.5)	7 (30.4)	3 (11.1)	3 (13.0)	7 (20.0)
≥2+	40 (37.0)	4 (17.4)	11 (40.7)	10 (43.5)	15 (42.9)
RAS blockade, n (%)*	15 (13.9)	7 (30.4)	4 (14.8)	1 (4.3)	3 (8.6)
Intravenous albumin administration, n (%)	12 (11.1)	1 (4.3)	3 (11.1)	1 (4.3)	7 (20.0)

**Use of Immunosuppressive drugs within 1 month of IST**					
Oral PSL, n (%)	107 (99.1)	23 (100.0)	26 (96.3)	23 (100.0)	35 (100.0)
Intravenous mPSL, n (%)*	28 (25.9)	5 (21.7)	2 (7.4)	10 (43.5)	11 (31.4)
Cyclosporine, n (%)	12 (11.1)	3 (13.0)	3 (11.1)	3 (13.0)	3 (8.6)
Rituximab, n (%)	1 (0.9)	0 (0.0)	1 (3.7)	0 (0.0)	0 (0.0)
**Cumulative incidence of remission and relapse**					
Remission, n (%)*	104 (96.3)	20 (87.0)	26 (96.3)	23 (100.0)	35 (100.0)
Remission within 6 months of IST, n (%)*	97 (89.8)	6 (54.5)	27 (96.3)	45 (93.8)	19 (90.5)
Relapse after remission, n (%)†	42 (43.3)	3 (50.0)	12 (44.4)	18 (40.0)	9 (47.4)

Mean±standard deviation; median (25%, 75%)

eGFR, estimated glomerular filtration rate; IST, immunosuppressive therapy; mPSL, methylprednisolone; PSL, prednisolone; RAS, renin-angiotensin system

*P <0.05 for analysis of variance, the Kruskal-Wallis test, the chi-square test, or the Fisher's exact test, as appropriately.

†Incidence of relapse in 97 patients with remission within 6 months of IST.
